# Extraneural and extracranial metastatic astrocytoma with primitive neuroectodermal component: a case report

**DOI:** 10.1186/s13256-025-05474-x

**Published:** 2025-09-30

**Authors:** Frayne Gomez, Stephen Evans, Forough Foroughi, Narayan Karanth

**Affiliations:** 1 The Canberra Hospital, Canberra, Australia; 2 Sullivan Nicolaides Pathology, Bowen Hills, Australia; 3 Royal Darwin Hospital, Darwin, Australia

**Keywords:** Astrocytoma, Primitive neuroectodermal tumor, Metastatic astrocytoma, Glioblastoma, Isocitrate dehydrogenase

## Abstract

**Background:**

Astrocytoma is a highly malignant tumor of the central nervous system with limited survival, despite standard multimodal therapies. While typically remains confined to the central nervous system, rare instances of extraneural and extracranial metastasis have been documented. The underlying pathophysiology remains poorly understood, with very few reported cases—particularly in tumors harboring isocitrate dehydrogenase mutations.

**Case presentation:**

We describe the case of a 21-year-old female New Zealander of European descent with O^6^-methylguanine-DNA methyltransferase promoter-methylated, IDH1-R132H-mutant astrocytoma (World Health Organization 2021 central nervous system grade 4) containing a primitive neuroectodermal tumor-like component, which exhibited diffuse skeletal and leptomeningeal metastases.

**Conclusion:**

This case represents an unusual presentation of extraneural and extracranial metastatic spread in a young female New Zealander of European descent with an isocitrate dehydrogenase-mutant astrocytoma. Compared with recent literature, it is notable for early systemic dissemination and the coexistence of bone and leptomeningeal disease. A multidisciplinary discussion concluded that additional biopsy of metastatic sites was unwarranted owing to clear pathological correlation, clinical decline, and poor prognosis. Molecular characteristics such as cyclin-dependent kinase inhibitor 2A/2B deletion may further refine classification. A review of current literature underscores the importance of the 2021 World Health Organization classification updates and highlights potential roles for epithelial–mesenchymal transition and glymphatic dissemination in metastasis. Targeted therapies—particularly isocitrate dehydrogenase inhibitors—are under active investigation. This case reinforces the need for ongoing research into predictive biomarkers and individualized treatment strategies.

## Introduction

Astrocytomas almost exclusively remain confined to the central nervous system (CNS), and systemic metastases are extremely rare, with an estimated incidence well below 1% (reported in < 0.5% of cases, comprising fewer than 200 published instances) [1]. The blood–brain barrier, absence of a true lymphatic network in the brain, and the typically short survival of patients are thought to limit hematogenous spread of these tumors. Nonetheless, rare cases of extraneural metastasis have been documented since the early twentieth century. In one comprehensive review up to 2011, Beauchesne *et al*. identified ~286 reported cases of gliomas with extraneural metastases [1]. Notably, > 80% of these occurred after neurosurgical interventions (for example. craniotomy or ventriculoperitoneal shunting), supporting the concept that disruption of protective CNS barriers or direct tumor seeding via shunts can facilitate escape of tumor cells into systemic circulation [1, 2]. Common sites of metastasis include the lungs/pleura (~60% of cases), lymph nodes (~51%, often cervical), bones (~31%), and liver (~22%) [1]. Osseous metastases, as seen in our patient, typically involve the vertebrae when they occur [1]. Overall, metastatic spread is especially uncommon in isocitrate dehydrogenase (IDH)-mutant gliomas—most reported extraneural metastases have involved IDH-wild-type tumors [3], meaning very few cases originate from an IDH-mutated subtype.

Within the spectrum of high-grade astrocytic tumors, there is a rare histopathological variant characterized by a primitive neuroectodermal tumor (PNET) component. Historically, such tumors were referred to as glioblastoma (GBM) with a PNET component. Patients with this variant tend to be younger, and some studies suggest the PNET-like component may confer an increased propensity for extracranial spread [4, 5]. Our patient’s case falls into this category. According to the latest World Health Organization (WHO) classification, the terminology for adult diffuse astrocytic tumors has been refined: an IDH-mutated diffuse astrocytoma with grade IV histological features is now termed “astrocytoma, IDH-mutant, WHO grade 4,” whereas the term “glioblastoma” is reserved exclusively for IDH-wild-type cases [6, 7]. This updated nomenclature is applied throughout our report in accordance with current guidelines.

In this context, we present a unique case of an IDH-1-R132H-mutant grade 4 astrocytoma with a PNET-like component that developed biopsy-proven extraneural metastases. The aim of this report is to highlight the occurrence of systemic metastasis in a diffuse astrocytoma variant and to align the discussion with current classification and management paradigms. This case has educational value for clinicians, underscoring that new systemic complaints in a patient with glioma (especially one who survives longer-term or has unusual histology) should prompt evaluation for metastasis despite its rarity.

## Case presentation

A fully independent 21-year-old female New Zealander of European descent presented to the emergency department with headache, dizziness, and vomiting. Cranial computed tomography (CT) and magnetic resonance imaging (MRI) demonstrated a large solitary mass in the left frontal lobe, abutting the falx cerebri and crossing the midline, measuring 6.2 × 6 × 6 cm. She underwent an urgent bifrontal craniotomy to debulk the tumor. Immediate postoperative CT scans demonstrated residual tumor in the left medial frontal lobe; therefore, a left redo frontal craniotomy for further debulking was performed. MRI of the brain 1 week later showed no residual disease in the left frontal region.

**Fig. 1:**
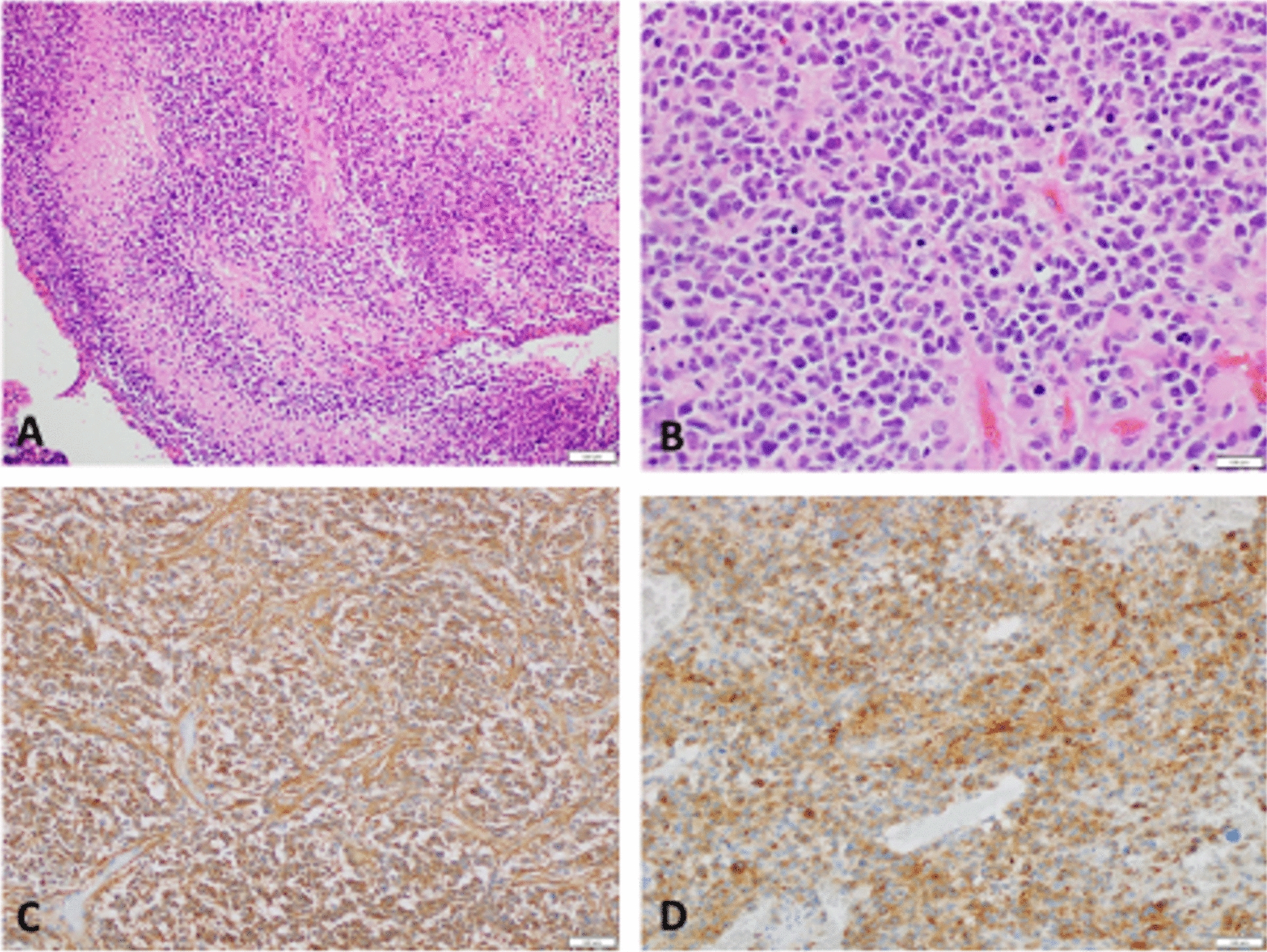
Histology from craniotomy. **A** High-grade astrocytoma showing predominantly small-blue-cell morphology with pseudopalisading necrosis and microvascular proliferation (Hematoxylin and Eosin). **B** Higher magnification showing marked cellularity with hyperchromatic pleomorphic nuclei ansd frequent mitoses (Hematoxylin and Eosin). **C**,  **D** Immunohistochemistry for isocitrate dehydrogenase-1 and synaptophysin demonstrating positive expression in tumor cells

Histopathology from both resections showed locally invasive high-grade glioma of gemistocytic morphology with areas of small-round-blue-cell appearance consisting of cells with small, hyperchromatic, pleomorphic nuclei and minimal cytoplasm set within a fibrillary background (Fig. 1). Mitotic figures were readily identifiable (> 50 mitoses per 10 high-power fields). There was extensive vascular proliferation with necrotic foci. Tumor cells were positive for glial fibrillary acidic protein (GFAP), oligodendrocyte lineage transcription factor 2 (Olig-2), tumor suppressor protein 53 (p53), and focally for synaptophysin but negative for neuronal nuclei antigen (NeuN) and alpha-thalassaemia/mental-retardation X-linked (ATRX). An IDH1-R132H mutation was present. Fluorescence *in-situ* hybridization demonstrated 19q deletion and 10q23 phosphatase and tensin homolog (PTEN) locus deletion. O^6^-methylguanine–DNA methyltransferase (MGMT) promoter methylation and epidermal growth factor receptor (EGFR) amplification were also identified. The proliferation index Kiel-67 (Ki-67) varied between 20–30%. These findings led to a final diagnosis of astrocytoma, IDH-mutant, WHO grade 4.

A course of adjuvant radiotherapy (60 Gray (Gy) in 30 fractions) was delivered with dexamethasone support. The patient declined concurrent and adjuvant temozolomide (per the Stupp protocol).

She was a New Zealander of European descent and had no significant past medical history; body-mass index was normal. Childhood history included bilateral tympanostomy tubes. She was a nonsmoker who consumed alcohol socially and was studying at university. Family history was notable for her mother’s colorectal cancer, now in remission.

**Fig. 2:**
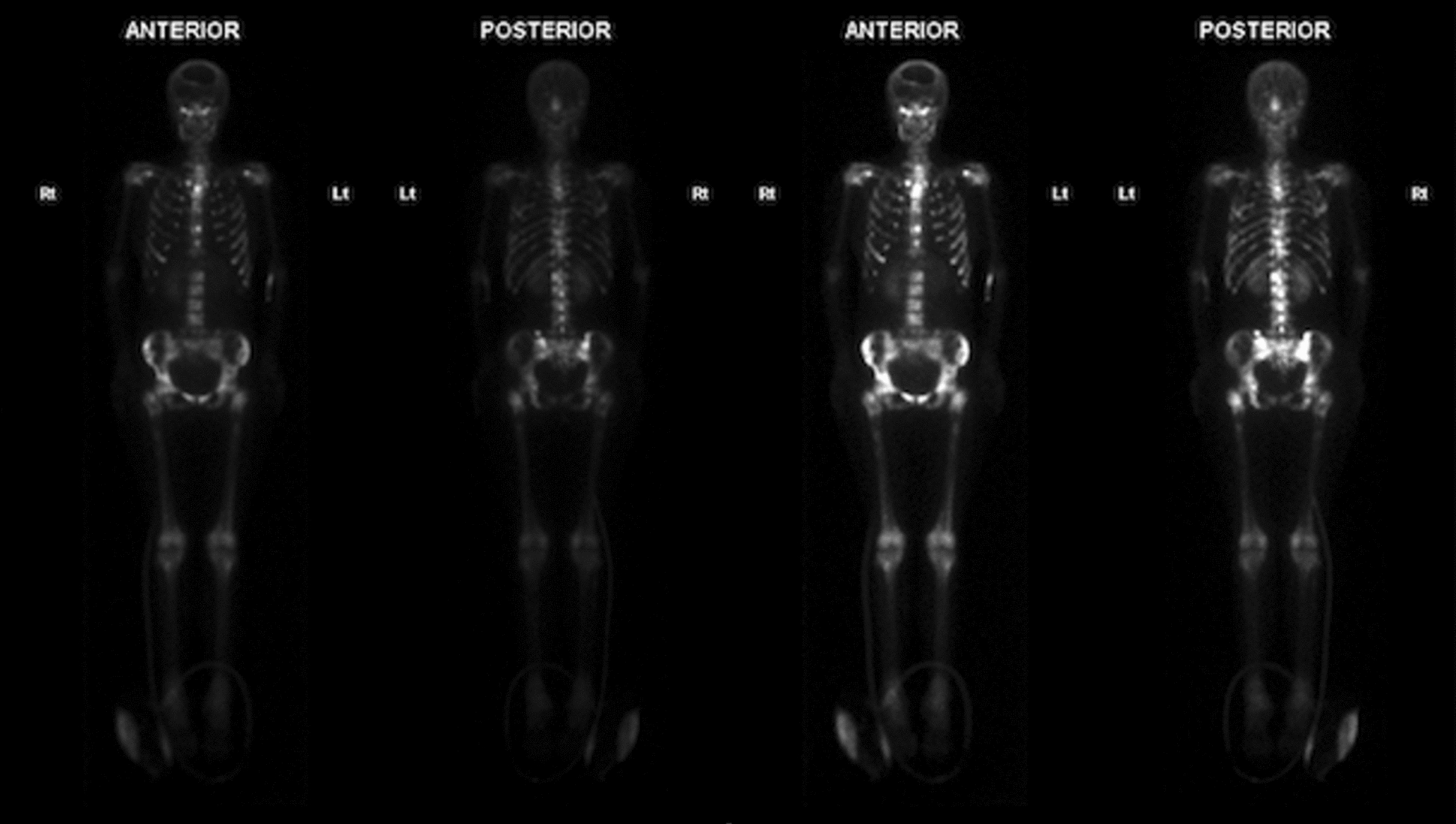
Whole-body technetium-99 nuclear-medicine scan showing diffuse metastatic bone disease with a primitive neuroectodermal tumor component

At 6 months after her original diagnosis, she represented with headache, new-onset back pain and seizures. Brain CT and MRI showed sizeable regrowth of residual tumor in the left frontal region with overlying leptomeningeal involvement. Contrast CT of the chest, abdomen, and pelvis demonstrated a patchy appearance of the thoracolumbar bone marrow. Single-photon emission computed tomography (SPECT) demonstrated high radiotracer uptake throughout the skeleton consistent with extensive metastatic bone disease, involving the skull, spine, rib cage, scapulae, clavicles, sternum, pelvis, and femora (Fig. 2).

A full blood count showed mild normocytic anemia and thrombocytopenia with a leuco-erythroblastic picture. Lumbar puncture revealed high protein, low glucose, and lymphocytosis; no organisms or malignant cells were detected, and flow cytometry was unremarkable. Leptomeningeal biopsy was not pursued owing to the risk of spinal-cord damage and the low likelihood of altering management.

**Fig. 3:**
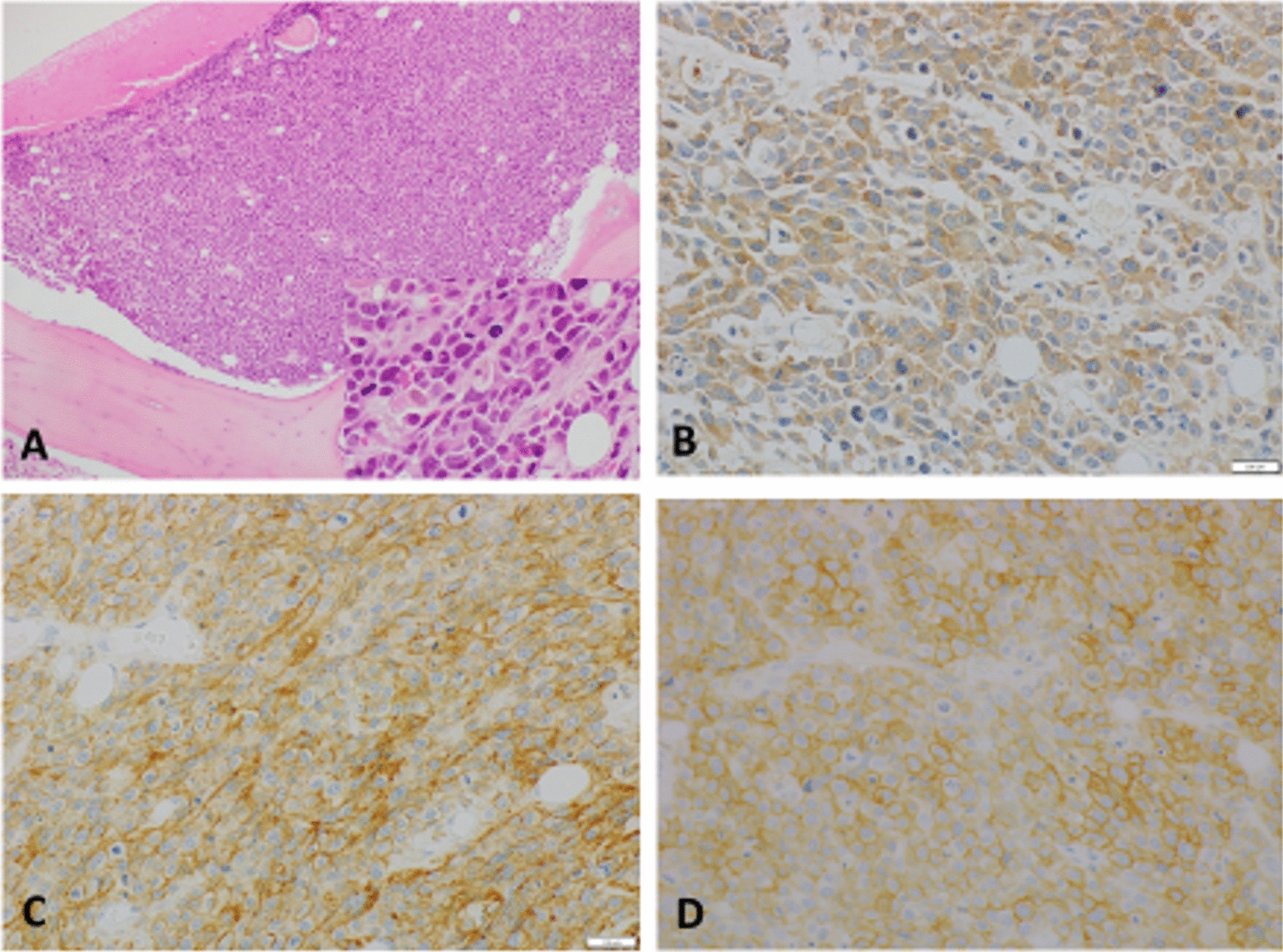
Bone-marrow biopsy. **A** Bone-marrow core showing diffuse infiltration by a small-round-blue-cell tumor morphologically similar to the primary brain tumor (inset: higher magnification). **B**–**D** Immunohistochemistry demonstrating tumor-cell positivity for isocitrate dehydrogenase-1, cluster of differentiation 56, and cluster of differentiation 117, respectively

Given the diffuse radiological bone and marrow involvement, a bone-marrow aspirate (dry tap) was performed (Fig. 2). This showed a primitive malignant tumor with small-round-blue-cell morphology and ~100% cellularity; normal hematopoiesis was virtually absent. Tumor cells were positive for tumor protein 53 (TP53), cluster of differentiation (CD)117, IDH1-R132H, synaptophysin, and CD56, but negative for avian myelocytomatosis viral oncogene homolog (C-MYC), CD3, CD10, CD20, CD34, CD38, CD45, CD79a, B-cell lymphoma 2 (BCL-2), glial fibrillary acidic protein (GFAP), AE1/AE3, Olig-2, and S100 calcium-binding protein B (S100). Ki-67 positivity was 20%. Nuclear staining for integrase interactor 1 (INI-1) was retained. Direct comparison with the original brain-tumor histology confirmed metastatic disease with a PNET component (Fig. 3).

With these findings, her condition was deemed terminal with a very poor prognosis. There was no role for further surgery, radiotherapy, or systemic therapy, and her Eastern Co-operative Oncology Group (ECOG) performance status was 3. She was referred to hospice care and died 8 months after the original diagnosis. Table 1 provides a summary of the patient's oncological journey.

**Table 1:**
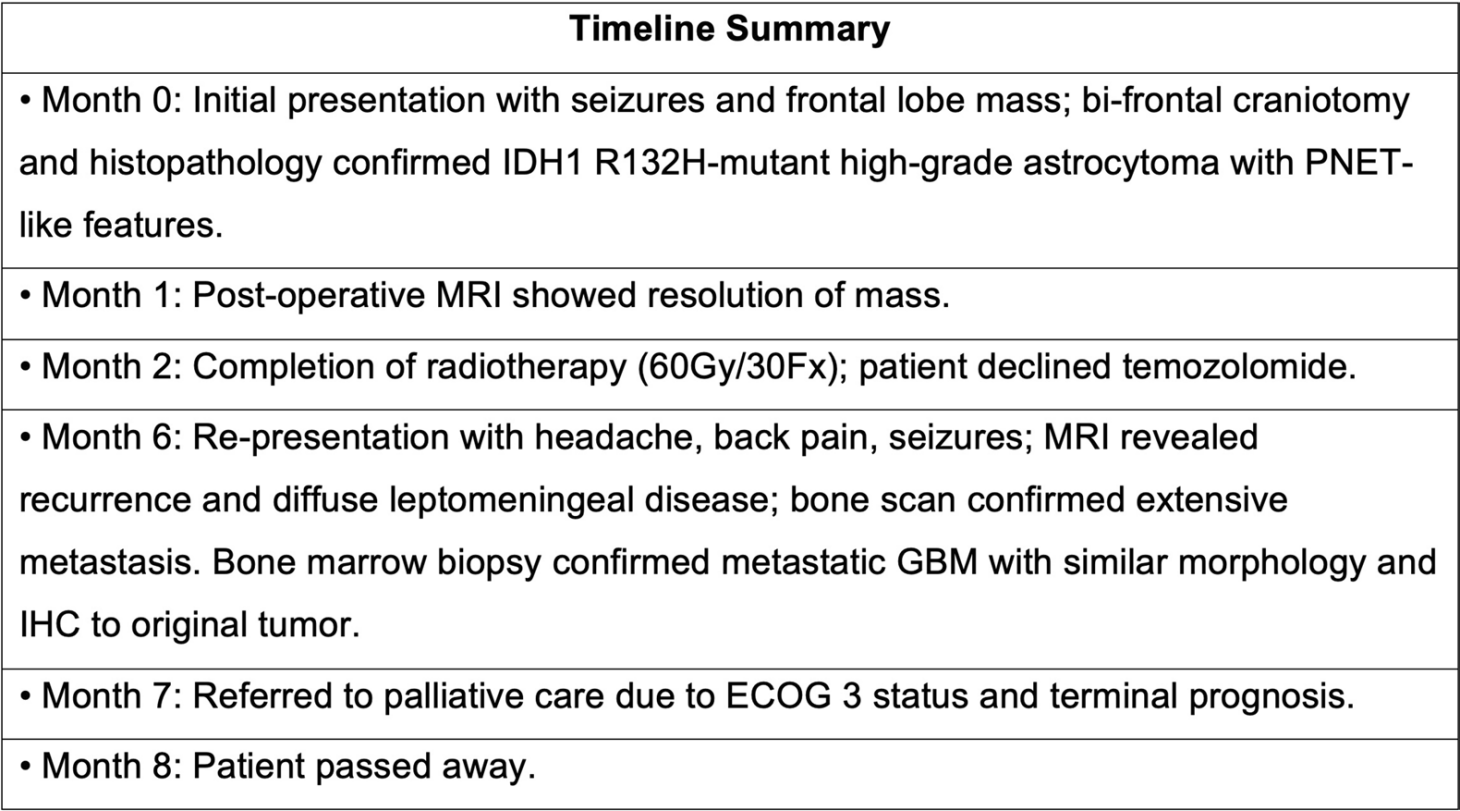
Timeline of diagnosis and treatment for our patient

## Discussion

Extraneural metastasis of diffuse gliomas—especially those with IDH mutations—is exceedingly rare. Systemic dissemination of glioblastoma (GBM) has an incidence of only ~0.2–0.5% [2, 8], and by 2020, only ~100 cases of extraneural osseous metastases had been documented [5]. Most reported metastatic GBMs are IDH-wild-type; these often exhibit mesenchymal features or gliosarcomatous differentiation, which are thought to enable greater invasiveness and vascular dissemination. Epithelial-to-mesenchymal transition (EMT) has been identified as a key mechanism promoting glioma cell invasiveness and metastatic potential [9]. In contrast, our report adds to the limited—yet growing—number of IDH-mutant astrocytomas with distant spread [10]. A recent single-institution series (*n* = 16) spanning 3 decades underscored how uncommon extracranial metastases are and was the first to perform next-generation sequencing of paired primary and metastatic glioma tissue [10]. Genomic analysis revealed shared and private mutations, with a predominance of tumor-suppressor-gene alterations (for example. PTEN loss, cyclin-dependent kinase inhibitor 2A/B (CDKN2A/B) deletion) in metastatic lesions [3].

A 2023 case report by Sudarsan *et al*. [11] described skeletal metastases in a 39-year-old with an IDH1-mutant astrocytoma (WHO grade 4) who remained alive following palliative chemotherapy and metastasis resection. In contrast, our patient experienced fulminant systemic progression, succumbing just 8 months from diagnosis. Unlike prior series—where most patients were male and metastasis followed multiple surgeries—our patient was a young female who developed extensive bony and leptomeningeal spread within 6 months, without shunting or postoperative complications. Contributors to dissemination include surgical manipulation, tumor proximity to meninges, and venous or lymphatic invasion [2]; however, none fully explain this fulminant course. The PNET-like component and upregulation of neuronal markers (synaptophysin, CD117) may indicate an aggressive clone with metastatic potential. Rong *et al*. reported a similar GBM-PNET variant in a 20-year-old man that disseminated to the axial skeleton within 7 months [4], supporting this hypothesis.

Recent literature has illuminated mechanisms by which gliomas overcome anatomic constraints: activation of EMT programmes, dissemination via cerebrospinal fluid and the glymphatic–meningeal–lymphatic pathway, and acquisition of genetic alterations conferring invasiveness (for example. PTEN loss, EGFR amplification, CDKN2A/B deletion) [3, 9, 12]. Although metastasis sequencing was not performed in our case, the primary-tumor profile (IDH1 mutation, TP53 mutation, ATRX loss, MGMT methylation) differs from the more typical metastasising GBM profile (IDH-wild-type with telomerase reverse transcriptase (TERT), EGFR, PTEN, CDKN2A/B alterations) [3]. This highlights the need for further research into the factors enabling aggressive behavior in IDH-mutant tumors.

Management of metastatic high-grade glioma remains palliative. Molecular profiling may identify actionable mutations: v-raf murine sarcoma viral oncogene homolog B1 (BRAF) V600E (for BRAF/MEK inhibitors) or fibroblast growth factor receptor–transforming acidic coiled-coil containing protein (FGFR–TACC) fusions (for FGFR inhibitors) in selected cases. In our patient, no such targets were present, but the IDH1 mutation offers a potential avenue. Mutant-IDH inhibitors (ivosidenib, olutasidenib, and vorasidenib) are under active investigation. The phase III “Investigating Non-enhancing Diffuse IDH-mutant Glioma Outcomes” (INDIGO) trial showed brain-penetrant IDH1/2 inhibition with vorasidenib prolonging progression-free survival in residual/recurrent IDH-mutant low-grade gliomas [13]. Early-phase studies in progressive high-grade disease show disease stabilization in a subset [13]. Unfortunately, our patient deteriorated before experimental IDH-directed therapy could be attempted. MGMT promoter methylation—normally predictive of temozolomide benefit—likely would not have overcome the aggressive metastatic course.

In view of clear histopathological concordance between intracranial and distant lesions and rapid clinical decline, further metastatic-site biopsy was eschewed. This aligned with the patient’s goals of care and the adopted palliative focus.

## Conclusion

This case highlights a rare presentation of extraneural, extracranial metastatic spread from an IDH-mutant astrocytoma (WHO grade 4) with PNET-like features. Despite the modern exclusion of the term “glioblastoma” for IDH-mutant tumors, aggressive behavior and widespread dissemination can still occur. Thorough molecular profiling and awareness of updated WHO criteria are essential. Clinicians should maintain vigilance for systemic metastasis when new symptoms arise, even in tumors traditionally considered nonmetastasising. Targeted therapies—particularly IDH inhibitors—show promise but require further study. Ongoing research should prioritize predictive biomarkers (for example. CDKN2A/B loss) and dissemination pathways to develop individualized treatments that improve survival and quality of life in this challenging scenario.

## Data Availability

We can provide support data if necessary, where applicable.
